# False discovery rates in spectral identification

**DOI:** 10.1186/1471-2105-13-S16-S2

**Published:** 2012-11-05

**Authors:** Kyowon Jeong, Sangtae Kim, Nuno Bandeira

**Affiliations:** 1 Department of Electrical and Computer Engineering, University of California San Diego, San Diego, CA, USA; 2 Department of Computer Science and Engineering, University of California San Diego, San Diego, CA, USA; 3 Skaggs School of Pharmacy and Pharmaceutical Sciences, University of California San Diego, San Diego, CA, USA; 4 Center for Computational Mass Spectrometry, University of California San Diego, San Diego, CA, USA

## Abstract

Automated database search engines are one of the fundamental engines of high-throughput proteomics enabling daily identifications of hundreds of thousands of peptides and proteins from tandem mass (MS/MS) spectrometry data. Nevertheless, this automation also makes it humanly impossible to manually validate the vast lists of resulting identifications from such high-throughput searches. This challenge is usually addressed by using a Target-Decoy Approach (TDA) to impose an empirical False Discovery Rate (FDR) at a pre-determined threshold *x*% with the expectation that at most *x*% of the returned identifications would be false positives. But despite the fundamental importance of FDR estimates in ensuring the utility of large lists of identifications, there is surprisingly little consensus on exactly how TDA should be applied to minimize the chances of biased FDR estimates. In fact, since less rigorous TDA/FDR estimates tend to result in more identifications (at higher 'true' FDR), there is often little incentive to enforce strict TDA/FDR procedures in studies where the major metric of success is the size of the list of identifications and there are no follow up studies imposing hard cost constraints on the number of reported false positives.

Here we address the problem of the accuracy of TDA estimates of empirical FDR. Using MS/MS spectra from samples where we were able to define a *factual *FDR estimator of 'true' FDR we evaluate several popular variants of the TDA procedure in a variety of database search contexts. We show that the fraction of false identifications can sometimes be over 10*× *higher than reported and may be unavoidably high for certain types of searches. In addition, we further report that the two-pass search strategy seems the most promising database search strategy.

While unavoidably constrained by the particulars of any specific evaluation dataset, our observations support a series of recommendations towards maximizing the number of resulting identifications while controlling database searches with robust and reproducible TDA estimation of empirical FDR.

## Introduction

Mass spectrometry (MS) based proteomics studies often generate millions of tandem mass spectra. These spectra are usually assumed to come from peptides and typically interpreted using a database search engine. There are numerous database search engines available such as SEQUEST [1], Mascot [2], X!Tandem [3], OMSSA [4], InsPecT [5] and MS-GFDB [6]. These engines take a set of spectra and a protein database as the input and output peptide-spectrum matches (PSMs) by scoring each spectrum against the peptides in the database and assigning the best-scoring peptide as a "match" to each spectrum. In most experiments, only a small portion of these PSMs (20% - 40%) represent plausible matches [5-7]. Therefore, identifying correct PSMs among a mixture of correct and incorrect PSMs is an important problem in MS based proteomics. Since confidence in PSM assignments is usually represented as a score, this problem is equivalent to setting up a score threshold where PSMs with scores above the threshold are regarded as *positive discoveries *(or positive PSMs) while the remaining are regarded as *negative discoveries *(or negative PSMs). The score threshold must be appropriately determined because low thresholds lead to excessive false positives and high thresholds lead to too many false negatives.

The target-decoy approach (TDA) [7,8] is currently the most widely used strategy to address this problem. Given a protein database (target database), this approach requires that spectra be searched not just against the target database but also against a *decoy *database. A decoy database is a reversed, shuffled (e.g., permuted) or otherwise randomized database of the same size as the target database. It is assumed that the positive PSMs from the decoy database (decoy PSMs) are false and that the expected number of decoy PSMs equals the expected number of false positive PSMs from the target database. Thus, by counting the number of decoy PSMs, one can estimate the *False Discovery Rate *(*FDR*) - the proportion of false PSMs among positive PSMs. Estimating FDRs via TDA is currently the standard in high-throughput MS studies because it is simple, easily implementable, and widely applicable to various experimental set-ups while successfully distinguishing correct and incorrect PSMs.

However, there is no consensus on the exact procedure for TDA - a worrisome situation since the quality of the resulting FDR estimates and the number of resulting PSMs are strongly dependent on such procedural variations. For example, there are multiple methods to generate decoy databases (e.g., it could be a reversed, shuffled or randomized version of the target database) but it remains an open problem to determine the optimal way of generating and using decoy databases. Also, it is questionable whether to search the target and the decoy database separately or to search the concatenated target and decoy databases. Furthermore, even after the score threshold is determined, it is ambiguous what formula to use to calculate the FDR. Because of all these "variations", the same FDR (e.g., FDR 1%) may mean a different confidence level depending on the specific procedure, and it is often difficult to determine how much trust can be allowed for FDRs reported in research papers on MS studies.

We compare various TDA procedures and assess them in terms of how accurate they estimate the "true" FDRs and how many PSMs they identify at a fixed true FDR. We also show how different database search parameters such as the the choice of the protein database, parent mass tolerance and enabling/disabling two-pass searches affect the accuracy of FDR estimation and the resulting set of PSMs. Based on our results, we recommend a set of TDA guidelines and search parameters towards improving the accuracy of FDR estimates while also producing more resulting PSMs.

We used X!Tandem [3] and MS-GFDB [6] as the database search engines. The conclusions presented here should apply to most other database search engines but may vary depending on particular implementation and design details.

## Materials

### MS/MS spectra

The main MS/MS spectra dataset used in this study was the LTQ-Orbitrap dataset in Mix 7 from the *ISB Standard Protein Mix Database *[9]. It consists of 47,292 spectra (denoted by **ISB-All**) from 10 replicates generated from tryptic digests of 18 proteins called *ISB Standard Protein Mix*. For most experiments, a subset containing 4,966 spectra from replicate 02 (denoted by **ISB-02**) were used.

We also analyzed the *Study 6 LTQ-XL-Orbitrap@86 *data set generated by the clinical proteomic technology assessment for cancer (CPTAC) network [10]. This dataset consists of LTQ-Orbitrap spectra from tryptic digests of yeast proteins with Sigma UPS1 spiked in. From the original dataset, we took 124,193 spectra to form **Y-All **dataset and further randomly selected 9,758 spectra out of **Y-All **dataset to form **Y-Small **dataset.

To compute factual FDRs (to be defined below), we additionally obtained a dataset of monoclonal antibody spectra from a previous protein sequencing study by Bandeira et al. [11] consisting of 19,982 spectra (denoted by **AB-All**). Among them, 6,319 Spectra from trypsin and chymotrypsin digests (denoted by **AB-TC**) were mainly used for most experiments.

### Protein database

We used the protein database of ISB Standard Protein Mix (18 proteins, 7,440 amino acids, denoted by **ISB**) for the ISB-All and ISB-02 datasets and the yeast database (from Ensembl ftp://ftp.ensembl.org, release 60, 6696 proteins, 3,011,992 amino acids, denoted by **Yeast**) for the Y-All and Y-Small datasets.

We also obtained an Arabidopsis thaliana database from the Arabidopsis Information Resource (TAIR) (http://arabidopsis.org, release 9, 33,410 proteins, 13,468,323 amino acids). The Arabidopsis thaliana database (denoted by **AT**) was also used to compute factual FDRs.

### Database search engine

We used X!Tandem (version 12/01/2011) [3] and MS-GFDB (version 01/06/2012) [6] as database search engines. For both engines, the parent mass tolerance was set to either 2.5 Da or 30 ppm (parts per million) according to the experiment (see Table 1). When the parent mass tolerance was 30 ppm, we allowed isotopic mass errors (i.e., +1, +2 and -1 Da errors) in the parent mass because such errors are very common for LTQ-Orbitrap spectra. We used the spectral probability for MS-GFDB and the hyper score for X!Tandem to score PSMs unless otherwise noted. Only the best match per spectrum was reported and no spectrum quality filter was used. For X!Tandem, the fragmentation ion tolerance was set to 0.5 Da and the two-pass search was deactivated.

**Table 1:** Details on experiments performed

Search#^1^	Spectra^2^	Database^3^	Decoy^4^	PMTol^8^	Note^11^
I-1	ISB-02	ISB	Rev^5^	2.5 Da^9^	
I-2	ISB-02	ISB	Shfl^6^	2.5 Da	
I-3	ISB-02	ISB+Yeast	Rev	2.5 Da	
I-4	ISB-02	ISB+Yeast	Shfl	2.5 Da	
I-5	ISB-02+AB-TC	ISB+Yeast	Rev	2.5 Da	
I-6	ISB-02+AB-TC	ISB+Yeast	Shfl	2.5 Da	
I-7	ISB-02+AB-TC	ISB+AT	Rev	2.5 Da	
I-8	ISB-02+AB-TC	ISB+AT	Shfl	2.5 Da	
I-9	ISB-02	ISB	Sep.Rev^7^	2.5 Da	
I-10	ISB-02+AB-TC	ISB+Yeast	Sep.Rev	2.5 Da	
I-11	ISB-02+AB-TC	ISB+AT	Sep.Rev	2.5 Da	
I-12	ISB-02	ISB+Yeast	Rev	2.5 Da	Alt.Formula^12^
I-13	ISB-02+AB-TC	ISB+Yeast	Rev	2.5 Da	Alt.Formula
I-14	ISB-02+AB-TC	ISB	Rev	2.5 Da	
I-15	ISB-02+AB-All	ISB+Yeast	Rev	2.5 Da	
I-16	ISB-02	ISB	Rev	30 ppm^10^	
I-17	ISB-02+AB-TC	ISB+Yeast	Rev	30 ppm	
I-18	ISB-02+AB-TC	ISB+AT	Rev	30 ppm	
I-19	ISB-02	ISB	Rev	2.5 Da	Alt.Score^13^
I-20	ISB-02+AB-TC	ISB+Yeast	Rev	2.5 Da	Alt.Score
I-21	ISB-02+AB-TC	ISB+AT	Rev	2.5 Da	Alt.Score
I-22	ISB-All+AB-TC	ISB+Yeast	Rev	2.5 Da	
I-23	ISB-All+AB-TC	ISB	Rev	2.5 Da	
Y-1	Y-Small+AB-TC	Yeast+AT	Rev	30 ppm	
Y-2	Y-Small+AB-TC	Yeast+AT	Shfl	30 ppm	
Y-3	Y-Small+AB-TC	Yeast+AT	Sep.Rev	30 ppm	
Y-4	Y-Small+AB-TC	Yeast+AT	Rev	30 ppm	Alt.Formula
Y-5	Y-Small+AB-TC	Yeast	Rev	30 ppm	
Y-6	Y-Small+AB-All	Yeast+AT	Rev	30 ppm	
Y-7	Y-Small+AB-TC	Yeast+AT	Rev	30 ppm	Alt.Score
Y-8	Y-All+AB-TC	Yeast+AT	Rev	30 ppm	
Y-9	Y-All+AB-TC	Yeast	Rev	30 ppm	
Y-10	Y-Small+AB-TC	Yeast+AT	Rev	30 ppm	TwoPass(1)^14^
Y-11	Y-Small+AB-TC	Yeast+AT	Rev	30 ppm	TwoPass(2)
Y-12	Y-Small+AB-TC	Yeast+AT	Rev	30 ppm	TwoPass(3)
Y-13	Y-Small+AB-TC	Yeast+AT	Rev	30 ppm	TwoPass(4)

For each of the searches I-1 to I-23 (Y-1 to Y-13), we counted the numbers of positive target PSMs (*N_target_*) at factual/empirical FDR 5% (1%) and computed the corresponding factual/empirical FDR of the positive PSMs. The underlined characters represent either dummy spectra, dummy databases, or dummy tolerance. ^1^Search identifier; ^2^MS/MS spectra used; ^3^Protein database; ^4^Decoy database type; ^5^Reversed decoy database; ^6^Shuffled decoy database; ^7^Separate search against target and reversed decoy database; ^8^Parent mass tolerance; ^9^Dalton; ^10^Parts per million; ^11^Additional note; ^12^Alternative formula was used to calculate FDR (see text); ^13^Alternative score was used to calculate FDR (see text); ^14^Two-pass searches (see text and Table 11).

## Methods

The most commonly used TDA procedure (denoted by **Standard TDA Protocol**) is as follows:

Given a set of spectra, a protein database (target database) and a database search engine,

1. Generate a decoy database by reversing the target database.

2. Concatenate the target and decoy database and run a database search engine against the concatenated database. For each spectrum, consider only the best scoring (either target or decoy) PSM.

3. Sort all PSMs in decreasing (or increasing) order of match scores (or E-values/p-values).

4. For a threshold *t*, estimate the FDR as *N_decoy_/N_target _*where *N_target _*(*N_decoy_*) is the number of positive target (decoy) PSMs (i.e., PSMs with scores better than *t*).

5. Report the set of target PSMs with scores better than *t *and a corresponding FDR.

Although the above TDA procedure is frequently used, many researchers do not follow exactly these steps. For example, instead of using the reversed database, some generate decoy databases by shuffling protein sequences in the target database [5] or enumerating amino acids randomly. Also, some prefer to run database searches separately for the target and decoy database and consider two PSMs per spectrum (one from the target database and the other from the decoy database). Moreover, some use 2 · *N_decoy_/*(*N_target _*+ *N_decoy_*) instead of *N_decoy_/N_target _*as the formula to compute FDRs. Depending on such choices, the set of resulting PSMs is very likely to vary.

In addition to the specific TDA procedure, one may get significantly different resulting PSMs depending on the choice of the protein database and search parameters. Below, we evaluate how these factors affect FDR estimation and change the resulting set of PSMs. In particular, we address the following cases/issues:

1. How to construct a decoy database: reversed vs shuffled

2. Concatenated vs separate decoy

3. Choice of formula to calculate FDR

4. Impact of the size of the database

5. How the number of spectra affects the results

6. Expected gains from accurate peptide parent masses

7. How the score normalization affects the results

8. PSM-level vs Peptide-level FDR

9. Two-pass searches and TDA

To address these issues, we designed a set of experiments by varying the set of spectra, protein database, TDA procedure, and search parameters (Table 1). For each experiment we measure how accurate the FDR estimation is by measuring the *factual FDR*. The *factual FDR *is defined as follows: If we are given a dataset where all spectrum identifications are perfectly known (a *fully-labeled approach*) in advance then one can easily validate the FDR estimated via TDA (denoted by *empirical *FDR) because it would be possible to compute the "true" FDR. But since such a dataset is not readily available, similar to Granholm et al. [12], we use a *semi-labeled approach *where false PSMs (termed *dummy *PSMs) are intentionally introduced using the following three ways.

1. Dummy databases: let *dummy proteins *be the proteins from which the searched spectra are not supposed to be generated. The *dummy database *is a database containing only dummy proteins. For example, consider the search of ISB-All spectra against ISB+Yeast (i.e., a database formed by concatenating ISB and Yeast databases) or ISB+AT database. We do not expect any significant match between the spectra in ISB-All and proteins in Yeast or AT databases. Thus, in this case, Yeast or AT databases are dummy databases for ISB-All dataset. All PSMs matched to dummy databases are dummy PSMs.

2. Dummy spectra: the *dummy spectra *are the spectra that are not supposed to be matched to the database searched against. For instance, we sometimes appended the spectra from either AB-TC or AB-All to ISB-02, ISB-All, Y-Small, or Y-All datasets and searched the merged datasets. Since we do not expect any significant match between the spectra in AB-All or AB-TC dataset and any protein sequence database used, AB-TC or AB-All spectra are dummy spectra for all experiments. All PSMs from dummy spectra are dummy PSMs.

3. Dummy parent mass tolerance: all spectra used in our experiments were obtained with a LTQ-Orbitrap, using an MS acquisition mode where the parent mass error is usually less than 30-50 ppm . Although running database searches with parent mass tolerance 50 ppm would be enough to find most correct matches, we used 2.5 Da parent mass tolerance (*dummy tolerance*) instead. Dummy parent mass tolerance was applied only to the experiments for the ISB-All and ISB-02 datasets. All PSMs with parent mass error larger than 50 ppm are dummy PSMs.

Note that all dummy PSMs are regarded as false but not all remaining PSMs (termed *putative *PSMs) are correct. To compute FDR (either empirical or factual) we have to estimate the number of false positive target PSMs. In case of empirical FDR, we estimate this number by the number of decoy PSMs (*N_decoy_*) without distinguishing between dummy and putative PSMs. In case of factual FDR, however, we use the information that positive dummy PSMs always represent false positive PSMs; the total number of false positive PSMs is thus the number of positive dummy PSMs (denoted by *N_dummy_*) plus the number of false positive PSMs among the putative PSMs (denoted by *N_false_*). Since *N_dummy _*is given, we only need to estimate *N_false_*.

To estimate *N_false_*, we use the Standard TDA Protocol. The inputs to the Standard TDA Protocol are the spectra of putative PSMs and the target database excluding any dummy proteins. The decoy database is generated by reversing this target database. For search, the parent mass tolerance is set to 50 ppm, and *N_false _*is given by the number of positive decoy PSMs in this search. The factual FDR is then defined by

$$\frac{N_{dummy}+\;N_{false}}{N_{target}}.$$

where *N_target _*denotes the number of positive target PSMs (including dummy PSMs). Since *N_false _*is estimated via TDA, the factual FDR also may suffer from the bias introduced by TDA as the empirical FDR does. However, since the factual FDR is using "extra information" of dummy PSMs (not available to the database search engine nor to TDA), it is expected to be closer to true FDR than empirical FDR in particular when the number of dummy PSMs (*N_dummy_*) is large. The definition of the factual FDR for two-pass searches is more complicated and is discussed below.

For each experiment, we fixed the factual/empirical FDR thresholds to 5% for searches I-1 to I-23 and 1% for searches Y-1 to Y-13 and reported the corresponding empirical/factual FDR values and the number of positive target PSMs (*N_target_*). Also for each experiment, we evaluated how significant the difference between empirical FDR and factual FDR is using the Fisher's exact test [13]. The 2*×*2 tables given in Table 2 were used for the Fisher's exact test. When the p-value of the Fisher's exact test (the Fisher's p-value) for a specific experiment was smaller than 5%, we regarded the empirical FDR for the experiment as inaccurate.

**Table 2:** 2 *×*2 tables for Fisher's exact test.

Estimator	# positives	# estimated false positives
FactFDR^1^	*N_target_*	*N_dummy _*+ *N_false_*
EmpiricalFDR^2^	*N_target_*	*N_decoy_*

FactFDR	*N_target _*+ *N_dummy _*+ *N_false_*	2 · *N_dummy _*+ *N_false_*
EmpiricalFDR	*N_target _*+ *N_decoy_*	2 · *N_decoy_*

FactPepFDR^3^	*N_target peptides_*	*N_dummy peptides _*+ *N_false peptides_*
EmpiricalFDR	*N_target_*	*N_decoy_*

FactPepFDR	*N_target peptides_*	*N_dummy peptides _*+ *N_false peptides_*
EmpiricalPepFDR^4^	*N_target peptides_*	*N_decoy peptides_*

When the p-value of the Fisher's exact test (the Fisher's p-value) for a specific experiment was smaller than 5%, we regarded the empirical FDR for the experiment as inaccurate. For most searches the definitions in (a) were used. The definitions in (b) were used only for the searches I-12, I-13, and Y-4 (i.e., searches using the alternative formula - see Table 5 and text). The definitions in (c) were used for experiments in Table 10 (empirical PSM-level FDR vs. factual peptide-level FDR), and the definitions in (d) were for experiments in Table 11 (empirical peptide-level FDR vs. factual peptide-level FDR). ^1^factual FDR; ^2^empirical FDR; ^3^factual peptide-level FDR; ^4^empirical peptide-level FDR; *N_target_*: the number of positive target PSMs; *N_dummy_*: the number of positive dummy PSMs; *N_false_*: the estimated number of false positive putative PSMs; *N_decoy_*: the number of positive decoy PSMs; *N_target peptides_*: the number of positive target peptides; *N_dummy peptides_*: the number of positive dummy peptides; *N_false peptides_*: the estimated number of false positive putative peptides; *N_decoy peptides_*: the number of positive decoy peptides.

Note that we do not aim to compare database search engines (i.e., MS-GFDB vs. X!Tandem). We only evaluate how FDR estimation via TDA is reliable and how the number of positive PSMs (or peptides) changes for different search strategies with different parameters or protocols.

## Results

### How to construct a decoy database: reversed vs shuffled

The decoy database can be generated by reversing the target proteins (reversed), shuffling amino acids of proteins (shuffled), or enumerating amino acids randomly (randomized) [7]. To avoid biased FDR estimates, it is important for decoy PSMs to have a score distribution similar to that of false target PSMs. To meet this condition, the decoy database should presumably preserve the amino acid composition (the numbers of individual amino acids) and the portion of shared peptides between different proteins in the target database. Additionally, for each spectrum, the number of target and decoy peptides matching the parent mass (within the chosen tolerance) should be similar.

The reversed database meets all these conditions when fully-tryptic peptide digestion is not enforced. Moreover, there is only one possible reversed database for every target database. This is beneficial because it removes the dependence on the randomization procedure and makes the FDR calculation deterministic and reproducible. Moreover, shuffled or randomized databases usually do not contain as many shared peptides (peptides that are shared between multiple proteins) as target protein database. This makes the actual search space in the decoy database larger than the search space in the target database, thus resulting in conservative FDR estimates [8]. Elias and Gygi noticed this problem and suggested a possible correction procedure [8] but most labs using shuffled databases still do not apply any correction.

To assess the impact of this choice of reversed vs shuffled decoy databases, we performed various pairs of searches (Table 3). For each pair of searches, the search conditions differ only in the use of decoy databases - reversed or shuffled. Except the databases, all searches followed the Standard TDA Procedure. Note that we did not apply the correction suggested by Elias and Gygi in the case of shuffled database search.

**Table 3:** Comparison between searches using reversed or shuffled decoy databases

Search#	Spectra	Database	PMTol	Decoy	EmpiricalFDR^1 ^fixed			FactFDR^2 ^fixed	
					
					$N_{target}^{3}$	FactFDR(%)	p-value(%) ^4^	*N_target_*	EmpiricalFDR(%)
I-1	ISB-02	ISB	2.5 Da	Rev	2329/1009	5.8/4.4	10.9/30.9	2279/1024	3.9/5.7
I-2	ISB-02	ISB	2.5 Da	Shfl	2339/1023	6.0/4.6	7.2/38.6	2279/1025	3.6/5.1

I-3	ISB-02	ISB+Yeast	2.5 Da	Rev	1578/602	4.7/5.1	40.8/50.3	1583/596	5.1/3.9
I-4	ISB-02	ISB+Yeast	2.5 Da	Shfl	1597/577	5.0/4.2	50.2/34.5	1589/588	4.8/5.6

I-5	ISB-02+AB-TC	ISB+Yeast	2.5 Da	Rev	1490/569	5.0/5.8	50.2/31.2	1480/553	4.5/4.3
I-6	ISB-02+AB-TC	ISB+Yeast	2.5 Da	Shfl	1488/530	5.0/4.0	50.2/28.7	1478/550	4.9/5.5

I-7	ISB-02+AB-TC	ISB+AT	2.5 Da	Rev	1320/441	4.6/4.1	36.6/38.0	1342/464	5.8/7.3
I-8	ISB-02+AB-TC	ISB+AT	2.5 Da	Shfl	1287/441	3.4/4.1	7.5/38.0	1342/464	6.8/7.3

Y-1	Y-Small+AB-TC	Yeast+AT	30 ppm	Rev	2574/1988	1.0/1.3	50.1/22.7	2588/1759	1.0/0.5
Y-2	Y-Small+AB-TC	Yeast+AT	30 ppm	Shfl	2554/1940	0.9/1.2	38.7/27.3	2620/1758	1.1/0.6

All searches followed the standard TDA procedure except the step 2 for shuffled database searches. The results in columns labeled "FDR fixed" are obtained at empirical FDR threshold of 5% (the searches I-1 to I-8) or 1%(the searches Y-1 to Y-2). The results in columns labeled "FactFDR fixed" are obtained at factual FDR threshold of 5% (the searches I-1 to I-8) or 1%(the searches Y-1 to Y-2). The underlined characters represent either dummy spectra, dummy databases, or dummy tolerance. The first numbers in *N_target_*/FactFDR/FDR/p-value fields are from MS-GFDB, and the second from X!Tandem. Note that we do not aim to compare database search engines (i.e., MS-GFDB vs. X!Tandem). We only evaluate how FDR estimation via TDA is reliable and how the number of positive PSMs (or peptides) changes for different search strategies with different parameters or protocols.

In contrast with popular belief, we did not observe a conservative estimation of FDR with shuffled decoy when compared to the reverse decoy database.

^1^the empirical FDR; ^2^the factual FDR; ^3 ^the number of positive target PSMs; ^4 ^Fisher p-value (see Table 2) - Fisher p-values less than 5% were emphasized with bold fonts.

#### Conclusion

No notable difference was observed between both approaches. Regardless of the database, both approaches reported similar numbers of PSMs at a fixed factual FDR (5% or 1%). The Fisher's p-value exceeded 5% for all cases; in contrast with popular belief, we did not observe a conservative estimation of FDR with shuffled decoy when compared to the reverse decoy database.

Based on these results, we would recommend the utilization of reversed decoy databases rather than shuffled decoy databases. While there was no noticeable disadvantage, there are several advantages of using reversed decoy databases: it is easy to generate, deterministic, reproducible and maintains the amino acid composition and distribution of shared peptides/parent masses between target and decoy databases.

### Concatenated vs separate decoy

Given target and decoy databases, it is common to concatenate them and search the concatenated database [8] but some groups prefer to search them separately [14]. The difference between the two approaches is whether to allow competition between target PSMs and decoy PSMs for every spectrum. The separated search does not allow this competition in that all positive decoy PSMs are considered for FDR calculation even if the same spectra of the PSMs match to the target database with better scores.

No competition in the separated searches means rather conservative FDR estimation because the fraction of false PSMs among all target PSMs is not counted (denoted by PIT in [14], but conventionally by π_0_) [14,15]. Several methods to estimate π_0 _were suggested (e.g., [16]), but we did not apply them for our experiments.

We compared both approaches using pairs of searches that differ only by the database search method - concatenated or separated search. For all searches, the standard TDA procedure was followed except step 2 for the separate search. For separate searches, the target and decoy databases are searched separately and the best scoring PSM is selected from each database and used for the empirical FDR calculation. Table 4 shows the results.

**Table 4:** Comparison between concatenated-decoy searches and separate-decoy searches

Search#	Spectra	Database	PMTol	Decoy	EmpiricalFDR fixed			FactFDR fixed	
					
					*N_target_*	FactFDR(%)	p-value(%)	*N_target_*	EmpiricalFDR(%)
I-1	ISB-02	ISB	2.5 Da	Rev	2329/1009	5.8/4.4	10.9/30.9	2279/1024	3.9/5.7
I-9	ISB-02	ISB	2.5 Da	Sep.Rev	2159/941	2.1/2.4	**0.0**/**0.4**	2287/1028	8.6/12.2

I-5	ISB-02+AB-TC	ISB+Yeast	2.5 Da	Rev	1490/569	5.0/5.8	50.2/31.2	1480/553	4.5/4.3
I-10	ISB-02+AB-TC	ISB+Yeast	2.5 Da	Sep.Rev	1462/504	4.6/3.6	40.3/18.7	1482/544	5.3/7.9

I-7	ISB-02+AB-TC	ISB+AT	2.5 Da	Rev	1320/441	4.6/4.1	36.6/38.0	1342/464	5.8/7.3
I-11	ISB-02+AB-TC	ISB+AT	2.5 Da	Sep.Rev	1287/453	3.4/4.9	**3.7**/56.1	1342/456	7.0/5.5

Y-1	Y-Small+AB-TC	Yeast+AT	30 ppm	Rev	2574/1988	1.0/1.3	50.1/22.7	2588/1759	1.0/0.5
Y-3	Y-Small+AB-TC	Yeast+AT	30 ppm	Sep.Rev	2501/1605	0.8/.0.7	33.1/28.7	2589/1759	1.4/1.5

All searches followed the standard TDA procedure except the step 2 for separate reverse database searches. The search I-9 demonstrates that the separate-decoy searches result in more conservative FDR estimation than concatenated-decoy searches, in particular for small databases.

#### Conclusion

The results show that the separate-decoy searches tend to estimate FDR conservatively. In particular for small databases, the separate-decoy searches resulted in more conservative FDR estimation than concatenated-decoy searches. For instance, the Fisher's p-value for the search I-9 was far less than 5% for both MS-GFDB and X!Tandem. This is because the π_0 _factor is expected to be smaller for small databases than for large databases.

Thus, we recommend to use concatenated-decoy search. Separate-decoy searches should be used with reliable estimation of the π_0 _factor, in particular for searches using small databases.

##### Choice of formula to calculate FDR

Given the numbers of target and decoy positive PSMs (denoted by *N_target _*and *N_decoy _*respectively), one can estimate FDR as *N_decoy_/N_target _*as in the standard TDA procedure. However, the first review on TDA by Elias and Gygi [8] suggested an alternative formula: 2 · *N_decoy_/*(*N_target _*+ *N_decoy_*) and both formulas are used in MS experiments (when using separate decoy with the π0 estimation, π_0 _· *N_decoy_/N_target _*should be used to estimate FDR, which is excluded because we are using concatenated decoy databases). The latter formula assumes the database search engine reports both target and decoy PSMs as positive discoveries. However, decoy PSMs do not need to be included in the final set of positive discoveries since these are obviously known to be false.

To compare how the choice of formula affects the results we modified the searches I-3, I-5, and Y-1 by changing the FDR formula (the searches I-12, I-13, and Y-4, respectively). For searches using the alternative formula, we used the second table in Table 2 for the Fisher's exact test. The comparison results are shown in Table 5.

**Table 5:** Comparison between two FDR formulas

Search#	Spectra	Database	PMTol	Formula	EmpiricalFDR fixed			FactFDR fixed	
					
					*N_target_*	FactFDR(%)	p-value(%)	*N_target_*	EmpiricalFDR(%)
I-3	ISB-02	ISB+Yeast	2.5 Da	1	1578/602	4.7/5.1	40.8/50.3	1583/596	5.1/3.9
I-12	ISB-02	ISB+Yeast	2.5 Da	2	1452/550	2.3/2.5	**0.0**/**2.9**	1583/596	9.6/7.4

I-5	ISB-02+AB-TC	ISB+Yeast	2.5 Da	1	1490/569	5.0/5.8	50.2/31.2	1480/553	4.5/4.3
I-13	ISB-02+AB-TC	ISB+Yeast	2.5 Da	2	1387/502	2.8/3.2	**0.6**/15.7	1480/553	8.7/8.3

Y-1	Y-Small+AB-TC	Yeast+AT	30 ppm	1	2574/1988	1.0/1.3	50.1/22.7	2588/1759	1.0/0.5
Y-4	Y-Small+AB-TC	Yeast+AT	30 ppm	2	2453/1626	0.8/0.8	27.8/36.2	2588/1759	2.0/1.0

The Formula field in the fifth column specifies the formula for the FDR calculation: 1 for *N_decoy_/N_target _*and 2 for 2 · *N_decoy_/*(*N_target_*+*N_decoy_*). For all searches, the standard TDA procedure was followed except the step 4 for searches using formula 2. The searches I-12 and I-13 show that using formula 2 results in conservative FDR estimation.

###### Conclusion

For most cases, the alternative formula (2 · *N_decoy_/*(*N_target _*+ *N_decoy_*)) resulted in conservative FDR estimation, yielding less positive target PSMs than the original formula *N_decoy_/N_target_*. For example, the Fisher's p-value was less than 5% in the search I-12 for both MS-GFDB and X!Tandem, indicating inaccurate FDR estimation from the alternative formula.

Since the FDR estimation of the original formula tends to be more accurate, we recommend using the original formula. In fact, recently Elias and Gygi also advocated using the original formula by stating that "decoy hits should not contribute to the final tally of incorrect hits since they can be easily recognized and removed" [17].

##### Impact of the size of the database

The choice of target database is obviously critical in all MS experiments. While this database should be chosen to include the sequences of proteins contained in the sample, it should also be as compact as possible because searching a larger database takes more time and more importantly reduces the number of resulting PSMs by allowing more choices for false PSMs. The former issue is well recognized by the community but the latter is often not addressed. Since larger databases increase the chances of false matches getting high-scores, the score threshold to determine positive PSMs at a fixed FDR also becomes higher for larger databases containing higher proportions of proteins not present in the sample.

To demonstrate the effect of database size, we ran searches against various databases of different sizes and compared the results (Table 6).

**Table 6:** Comparison between searches against databases of different sizes

Search#	Spectra	Database	PMTol	DB size	EmpiricalFDR fixed			FactFDR fixed	
					
					*N_target_*	FactFDR(%)	p-value(%)	*N_target_*	EmpiricalFDR(%)
I-1	ISB-02	ISB	2.5 Da	7,440	2329/1009	5.8/4.4	10.9/30.9	2279/1024	3.9/5.7
I-3	ISB-02	ISB+Yeast	2.5 Da	3,019,432	1578/602	4.7/5.1	40.8/50.3	1583/596	5.1/3.9

I-14	ISB-02+AB-TC	ISB	2.5 Da	7,440	2262/984	5.8/4.5	5.7/34.5	2221/995	4.0/6.0
I-5	ISB-02+AB-TC	ISB+Yeast	2.5 Da	3,019,432	1490/569	5.0/5.8	50.2/31.2	1480/553	4.5/4.3
I-7	ISB-02+AB-TC	ISB+AT	2.5 Da	13,475,763	1320/441	4.6/4.1	36.6/38.0	1342/464	5.8/7.3

Y-5	Y-Small+AB-TC	Yeast	30 ppm	3,011,992	3340/2734	1.2/1.0	30.0/50.1	3209/2717	0.8/1.0
Y-1	Y-Small+AB-TC	Yeast+AT	30 ppm	16,480,315	2574/1988	1.0/1.3	50.1/22.7	2588/1759	1.0/0.5

As expected, for smaller databases, TDA yielded more resulting PSMs. Fisher p-values were higher than 5% for all cases, which indicates that the FDR estimation via empirical FDR is reliable regardless of the database size.

###### Conclusion

As expected, for smaller databases, TDA yielded more resulting PSMs. The FDR estimation via empirical FDR was reliable regardless of the database size.

Based on these results, we recommend choosing the smallest possible database containing the sequences of proteins presumed to be in the sample.

##### How the number of spectra affects the results

In most high-throughput MS experiments, only less than 40% of all MS/MS spectra are identified. The remaining spectra are not identified because of reasons such as signal-to-noise ratio, poor peptide fragmentation, non-peptide spectra, spectra from peptides missing from the target database, post-translational modifications that are not considered in the database search, etc. If such unidentifiable spectra could be removed in advance, this would reduce the database search time and possibly produce more PSMs because unidentifiable spectra can only generate false PSMs and could thus increase the TDA-determined score threshold. To estimate the effect of unidentifiable spectra, we compared searches with various datasets differing only in the portion of unidentifiable spectra (Table 7).

**Table 7:** Comparisons between searches with different portions of unidentifiable spectra

Search#	Spectra	Database	PMTol	# spec	EmpiricalFDR fixed			FactFDR fixed	
					
					*N_target_*	FactFDR(%)	p-value(%)	*N_target_*	EmpiricalFDR(%)
I-1	ISB-02	ISB	2.5 Da	4,966	2329/1009	5.8/4.4	10.9/30.9	2279/1024	3.9/5.7
I-14	ISB-02+AB-TC	ISB	2.5 Da	11,285	2262/984	5.8/4.5	5.7/34.5	2221/995	4.0/6.0

I-3	ISB-02	ISB+Yeast	2.5 Da	4,966	1578/602	4.7/5.1	40.8/50.3	1583/596	5.1/3.9
I-5	ISB-02+AB-TC	ISB+Yeast	2.5 Da	11,285	1490/569	5.0/5.8	50.2/31.2	1480/553	4.5/4.3
I-15	ISB-02+AB-All	ISB+Yeast	2.5 Da	24,948	1367/531	4.2/5.3	33.0/34.5	1393/518	5.5/4.2

Y-1	Y-Small+AB-TC	Yeast+AT	30 ppm	16,077	2574/1988	1.0/1.3	50.1/22.7	2588/1759	1.0/0.5
Y-6	Y-Small+AB-All	Yeast+AT	30 ppm	29,740	2238/1913	1.1/1.4	44.2/14.7	2208/1629	0.9/0.7

Adding unidentifiable spectra reduces the number of positive PSMs, but does not change the accuracy of FDR estimations significantly.

###### Conclusion

Adding unidentifiable spectra reduces the number of positive PSMs, but does not change the accuracy of FDR estimations significantly. Thus filtering noisy spectra prior to a database search [18-20] should be helpful towards increasing the number of resulting identifications.

##### Expected gains from accurate peptide parent masses

Modern mass spectrometry instruments (e.g., FT/ICR or Thermo LTQ Orbitrap) can measure masses very accurately and are commonly configured to generate high-accuracy MS spectra (e.g., ≤50 ppm) and low-accuracy MS/MS spectra (e.g., ≤0.5 Da) [21]. The availability of high-accuracy parent masses allows database search engines to greatly restrict the masses of eligible database peptides and thus significantly reduces the number of peptides scored against each spectrum. Here we measured how the availability of high-accuracy parent masses changes the results (Table 8).

**Table 8:** Comparison between searches with strict and loose parent mass tolerance.

Search#	Spectra	Database	PMTol	EmpiricalFDR fixed			FactFDR fixed	
				
				*N_target_*	FactFDR(%)	p-value(%)	*N_target_*	EmpiricalFDR(%)
I-1	ISB-02	ISB	2.5 Da	2329/1009	5.8/4.4	10.9/30.9	2279/1024	3.9/5.7
I-16	ISB-02	ISB	30 ppm	2128/1009	N/A^1^	N/A	N/A	N/A

I-5	ISB-02+AB-TC	ISB+Yeast	2.5 Da	1490/569	5.0/5.8	50.2/31.2	1480/553	4.5/4.3
I-17	ISB-02+AB-TC	ISB+Yeast	30 ppm	1638/569	6.4/5.3	**3.1**/45.2	1570/565	3.9/4.8

I-7	ISB-02+AB-TC	ISB+AT	2.5 Da	1320/441	4.6/4.1	36.6/38.0	1342/464	5.8/7.3
I-18	ISB-02+AB-TC	ISB+AT	30 ppm	1358/463	3.2/5.1	**2.0**/50.3	1425/463	6.5/4.3

As expected, when using strict parent mass tolerance more PSMs were identified (at the same factual FDR threshold) in most cases.

^1 ^For the search I-16, the factual FDR is not available because no dummy element is used.

###### Conclusion

As expected, when using strict parent mass tolerance more PSMs were identified (at the same factual FDR threshold) in most cases. For the searches I-17 and I-18, the empirical FDRs reported by MS-GFDB were rather inaccurate. However, while the empirical FDR in I-17 was too conservative, that in I-18 was too liberal. This indicates that the empirical FDR in searches using strict tolerance is not strongly biased toward one direction. Thus, we recommend using strict tolerance in database searches.

##### How the score normalization affects the results

TDA implicitly assumes that given two PSMs (*S*_1_, *P*_1_) and (*S*_2_, *P*_2_) where *S*_1 _≠ *S*_2_, if *Score*(*S*_1_, *P*_1_) ≥ *Score *(*S*_2_, *P*_2_), the chances of (*S*_1_, *P*_1_) being correct should be higher than the chances of (*S*_2_, *P*_2_) being correct (namely, (*S*_1_, *P*_1_) is *better *than (*S*_2_, *P*_2_)). However, this is not true for all scoring functions. For example, SEQUEST Xcorr tends to assign large scores to long peptides. Thus, even if *Score*(*S*_1_, *P*_1_) ≥ *Score *(*S*_2_, *P*_2_), if *Length*(*P*_1_) *>> Length*(*P*_2_), it is possible that (*S*_1_, *P*_1_) is a worse match than (*S*_2_, *P*_2_). This *score normalization *problem is an important issue for TDA to work effectively.

Using probabilistic scores (e.g. q-value, p-value or posterior error probability) is a good solution to obtain a good normalization. Most database search engines nowadays report a pair of scores: a "raw" score and a probabilistic score. For example, Mascot reports ion scores and E-values and MS-GFDB reports MS-GF score and spectral probability. Alternatively, one can get probabilistic scores by running post-processing tools like Peptide-Prophet [22].

To estimate the effect of score normalization, we ran pairs of MS-GFDB searches. For each pair of searches, one used the spectral probability (probabilistic score) and the other used the MS-GF score (raw score) to compute FDR. The spectral probability can be considered simply as "better normalized" score of the MS-GF score for this experiment [23]. Table 9 shows the results.

**Table 9:** Comparison between searches with differently normalized scoring functions

Search#	Spectra	Database	PMTol	Score	EmpiricalFDR fixed			FactFDR fixed	
					
					*N_target_*	FactFDR(%)	p-value(%)	*N_target_*	EmpiricalFDR(%)
I-1	ISB-02	ISB	2.5 Da	SpecProb^1^	2329	5.8	10.9	2279	4.4
I-19	ISB-02	ISB	2.5 Da	MSGFRaw^2^	2079	4.6	36.5	2079	4.6

I-5	ISB-02+AB-TC	ISB+Yeast	2.5 Da	SpecProb	1490	5.0	50.2	1480	4.5
I-20	ISB-02+AB-TC	ISB+Yeast	2.5 Da	MSGFRaw	1272	5.7	25.2	1210	4.5

I-7	ISB-02+AB-TC	ISB+AT	2.5 Da	SpecProb	1320	4.6	36.6	1342	5.8
I-21	ISB-02+AB-TC	ISB+AT	2.5 Da	MSGFRaw	987	3.9	37.3	1064	6.1

Y-1	Y-Small+AB-TC	Yeast+AT	30 ppm	SpecProb	2574	1.0	50.1	2588	1.0
Y-7	Y-Small+AB-TC	Yeast+AT	30 ppm	MSGFRaw	1215	1.9	**1.8**	861	0.3

The spectral probability can be considered simply as "better normalized" score of the MS-GF score for this experiment [23]. Using the well-normalized score (i.e., the spectral probability) always produces substantially more resulting PSMs, with higher gains for larger databases. Furthermore, as in the search Y-7, the TDA-determined empirical FDR tended to be more accurate when well-normalized score was used.

^1^Spectral probability was used to compute the FDR; ^2^MS-GF score was used to compute the FDR.

###### Conclusion

Using the well-normalized score (i.e., the spectral probability) always produces substantially more resulting PSMs, with higher gains for larger databases. Furthermore, as in the search Y-7, the TDA-determined empirical FDR tended to be more accurate when well-normalized score was used. Thus, we recommend to use well-normalized scoring function (e.g., probability scores) to maximize the number of positive target PSMs at a fixed FDR. To compute FDRs separately depending on the precursor charge is also recommended if the scoring function is not well normalized across the spectra of different precursor charges. For example, most engines using peptide sequence tags (e.g., InsPecT [5]) identify spectra of charge 2 relatively well but struggle in identifying spectra with precursor charges 3 or more. For such database search engines, it is better to compute FDRs separately depending on the precursor charge to maximize the resulting PSMs (In fact, the script to compute FDRs contained in the InsPecT package computes FDR separately for charge 2 spectra and others).

##### PSM-level vs Peptide-level FDR

In MS experiments, it is common to compute FDRs at the PSM-level (as the portion of false PSMs among positive PSMs), and use the resulting PSMs to identify peptides (if at least one PSM is identified as peptide *P *then *P *is said to be identified). These identified peptides are in turn used to identify proteins (e.g. two-peptide rule: for a protein, if it contains at least two identified peptides, it is assumed to be identified). However, while multiple correct PSMs often correspond to a single correct peptide, false PSMs typically correspond to distinct false peptides. Consequently, even a set of PSMs with a very low (PSM-level) FDR may result in excessive false peptide identifications.

Computing the empirical peptide-level FDR is a readily-available solution to this problem: if multiple PSMs are matched to the same peptide, only the best-scoring PSM is retained; the peptide-level FDR is then calculated using only these best-scoring PSMs per peptide. The factual peptide-level FDR is defined similarly.

To demonstrate the problem of PSM-level FDRs, we reported factual peptide-level FDRs for various searches when the score threshold was determined using empirical PSM-level FDR (Table 10). Among the searches in Table 10, the search I-23 illustrates the problem most explicitly. The ISB-All dataset used in the search I-23 contains spectra of 10 replicate runs of the same ISB standard protein mixture and thus many spectra are expected to be identified as the same peptides. The factual peptide-level FDRs of this search were 42.8% and 39.6% for MS-GFDB and X!Tandem, respectively.

**Table 10:** Comparison between peptide-level factual FDR and PSM-level FDR

				EmpiricalFDR fixed			
				
Search#	Spectra	Database	PMTol	*N_target_*	# peptides^1^	FactPepFDR(%)^2^	p-value(%)
I-5	ISB-02+AB-TC	ISB+Yeast	2.5 Da	1490/569	600/262	12.0/11.8	**0.0**/**0.1**
I-22	ISB-All+AB-TC	ISB+Yeast	2.5 Da	13441/5086	1375/538	38.6/38.1	**0.0**/**0.0**

I-14	ISB-02+AB-TC	ISB	2.5 Da	2262/984	815/361	13.1/10.5	**0.0**/**0.1**
I-23	ISB-All+AB-TC	ISB	2.5 Da	19501/8497	1628/556	42.8/39.6	**0.0**/**0.0**

Y-1	Y-Small+AB-TC	Yeast+AT	30 ppm	2574/1988	2355/1841	1.1/1.4	37.8/15.8
Y-8	Y-All+AB-TC	Yeast+AT	30 ppm	9005/6269	3567/2640	2.4/2.0	**0.0**/**0.0**

Y-5	Y-Small+AB-TC	Yeast	30 ppm	3340/2734	3033/2515	1.2/1.0	19.1/54.5
Y-9	Y-All+AB-TC	Yeast	30 ppm	11151/8969	4341/3582	2.3/2.3	**0.0**/**0.0**

Score thresholds were determined using PSM-level FDR thresholds and used to calculate factual peptide-level FDRs. The results illustrate that PSM-level empirical FDR underestimates peptide-level FDR significantly (e.g., the searches I-22 and I-23).

^1^Number of distinct peptides; ^2^Factual peptide-level empirical FDR.

Table 11 shows the comparison between empirical peptide-level FDRs and factual peptide-level FDRs. For this experiment, the Fisher's exact tests were done using number of distinct peptides instead of PSMs.

**Table 11:** Comparison between peptide-level factual FDR and peptide-level FDR

				EmpiricalPepFDR^1 ^fixed					FactPepFDR fixed	
				
Search#	Spectra	Database	PMTol	*N_target_*	# peptides^1^	FactPepFDR(%)	p-value(%)	*N_target_*	# peptides	EmpiricalPepFDR(%)
I-5	ISB-02+AB-TC	ISB+Yeast	2.5 Da	1340/498	532/234	5.3/6.4	45.0/22.6	1335/479	529/226	4.9/4.4
I-22	ISB-All+AB-TC	ISB+Yeast	2.5 Da	10245/3688	758/304	6.6/6.6	7.0/**4.3**	9448/3596	696/292	2.4/4.1

I-14	ISB-02+AB-TC	ISB	2.5 Da	2088/907	727/333	6.7/5.1	10.4/50.3	1994/900	693/332	2.9/4.8
I-23	ISB-All+AB-TC	ISB	2.5 Da	16602/6676	1083/416	9.2/8.9	**0.0**/**2.0**	15663/6203	1015/385	1.7/1.3

Y-1	Y-Small+AB-TC	Yeast+AT	30 ppm	2574/1963	2355/1818	1.1/1.3	38.7/17.6	2556/1759	2339/1636	0.9/0.6
Y-8	Y-All+AB-TC	Yeast+AT	30 ppm	7867/5068	3142/2201	1.3/1.0	6.1/50.1	7121/5068	2849/2201	0.4/1.0

Y-5	Y-Small+AB-TC	Yeast	30 ppm	3209/2666	2916/2455	1.0/0.9	44.7/50.1	3209/2734	2916/2515	0.9/1.0
Y-9	Y-All+AB-TC	Yeast	30 ppm	10005/7309	3885/2987	1.0/0.9	50.0/44.9	10005/7309	3885/2987	0.9/0.9

Score thresholds were determined using empirical/factual peptide-level FDR and used to calculate factual/empirical FDRs. For the searches I-5, I-22, I-14, and I-23, the peptide-level FDR thresholds were set to 5%, and for the remaining searches they were set to 1%. The search I-23 illustrates the difficulty of enforcing peptide-level FDR when searching small databases.

^1^Empirical peptide-level FDR.

###### Conclusion

PSM-level FDR differs significantly from peptide-level FDRs. In particular when the larger datasets (e.g., ISB-ALL+AB-TC or Y-All+AB-TC) were used, the resulting empirical PSM-level FDRs seriously underestimated the factual peptide-level FDRs (up to 10 folds), indicating that the peptide-level FDR is more important when large datasets/experiments are considered. On the other hand, Table 11 demonstrates that in most cases the empirical peptide-level FDR reliably estimates the peptide-level FDR even if for some cases (e.g., the search I-23) the estimation was still too liberal.

Thus, in MS experiments where peptide identifications are used in downstream applications (e.g., protein identification) peptide-level FDR should be used instead of PSM-level FDR. Other applications choosing to use empirical PSM-level FDR should be required to present supporting evidence that such FDR estimates are accurate and appropriate for the proposed goals.

##### Two-pass searches and TDA

Craig and Beavis [24] pioneered the two-pass search approach that searches the target database twice. In the first pass, spectra are searched against the database to identify candidate proteins; in the second pass, spectra are again searched against only the candidate proteins identified in the first pass. The spectra matched in the first pass are sometimes removed in the second pass (matched spectrum removal (MSR) step [24,25]). This approach was originally proposed to accelerate the database search by quickly finding proteins containing non-modified fully tryptic peptide matches in the first pass and identifying more complex peptides (e.g., nontryptic peptides or peptides with modifications) in the second pass. In addition to expediting the database search, the two-pass approach can also be used to produce more resulting PSMs by reducing the database size in the first pass.

Recently, it was recognized that TDA should be carefully applied when estimating FDRs for two-pass searches [25-27]. Traditionally, TDA treats a database search engine as a black box that reports a sorted list of PSMs. If we consider a database search engine supporting the two-pass search (e.g., X!Tandem [3]) as a black box and apply TDA, the candidate proteins selected at the first pass will contain more target proteins than decoy proteins. Therefore, in the second pass, the assumption of TDA that matches to decoy are representative of false matches to target no longer holds and TDA will report a significantly smaller FDR than the true FDR. Results from the searches Y-10 and Y-11 in Table 12 illustrate this problem. When the empirical FDR was fixed to 1%, the factual FDRs of both searches were close to or exceeded 10%.

**Table 12:** Comparison between the single-pass search (the search Y-1) and various two-pass search methods (the searches Y-10 to Y-13)

Search#	Spectra	Database	PMTol			MSR^2^	EmpiricalFDR fixed			FactFDR fixed	
							
				IsTwoPass	2th decoy^1^		*N_target_*	FactFDR(%)	p-value(%)	*N_target_*	EmpiricalFDR(%)
Y-1	Y-Small+AB-TC	Yeast+AT	30 ppm	No	Rev	N/A	2574/1988	1.0/1.3	50.1/22.7	2588/1759	1.0/0.5

Y-10	Y-Small+AB-TC	Yeast+AT	30 ppm	Yes	Trad^3^	No	5361/5744	15.9/20.1	**0.0**/**0.0**	3260/2655	0.6/0.3
Y-11	Y-Small+AB-TC	Yeast+AT	30 ppm	Yes	Trad	Yes	4114/3925	7.3/10.1	**0.0**/**0.0**	3102/2320	0.9/0.5
Y-12	Y-Small+AB-TC	Yeast+AT	30 ppm	Yes	BK^4^	No	3529/3089	1.2/1.0	24.9/45.0	3262/3074	0.7/0.9
Y-13	Y-Small+AB-TC	Yeast+AT	30 ppm	Yes	BK	Yes	3137/2514	1.1/1.1	40.1/39.3	3103/2521	1.0/0.8

For the searches Y-10 and Y-11, the traditional second pass decoy database was used to estimate FDR (see text). For the searches Y-12 and Y-13, the decoy database proposed by Bern et al. [25] was used. Also, for the searches Y-11 and Y-13, the matched spectrum removal (MSR) step was used.

Low Fisher p-values in Y-10 and Y-11 illustrate that using the traditional second pass decoy database results in significant underestimation of the true FDR.

^1^The decoy database for the second pass search; ^2^Whether the matched spectrum removal step was used; ^3^The traditional decoy database; ^4^The BK decoy database.

To remedy this problem, Everett et al. [27] suggested to generate a decoy database for the second pass by reversing the candidate target proteins selected in the first pass. In this way, target and decoy databases in the second pass can have the same number of proteins. However, Bern and Kil [25] claimed that these target and decoy databases still can be "unbalanced" because the false positive PSMs in the target database are likely to have better scores than the positive decoy PSMs in the decoy database. They proposed to generate the decoy database by first taking candidate decoy protein sequences and second appending reversed sequences of candidate target protein sequences until the number of proteins in the decoy database equals to the number of the target proteins. The decoy database constructed in this way is specified by *BK decoy database*. On the other hand, the decoy database constructed by retaining only candidate decoy protein sequences is specified by *traditional decoy database*.

We tested two methods - the traditional and the BK decoy database - with or without the MSR step (we did not test the decoy database proposed by Everett et al. [27]). For this experiment, only the searches using Y-All or Y-Small databases were tested because the ISB database contains too few proteins to observe the effect of the reduced target database in the second pass. From the first pass search, we used score threshold corresponding to 1% empirical FDR to find candidate proteins.

For two-pass searches, the number of dummy PSMs (*N_dummy_*) can be counted as previously described (in Methods section), but the number of false positives out of putative PSMs (*N_false_*) should be estimated differently because the search space of a two-pass search is typically different from a single-pass search. We call the estimation method of *N_false _*for single-pass searches described in Methods section the *single-pass estimation method*.

To estimate *N_false _*for two-pass searches, first consider the cases in which the MSR step is not used. In this case, the search space is decided by the candidate proteins found in the first pass. To estimate *N_false_*, for each search we take the candidate proteins found in the first pass of the search, remove dummy proteins, and generate the BK decoy database using these proteins. The spectra excluding dummy spectra are searched against the target proteins (with dummy proteins removed) and the proteins in the generated BK decoy database. *N_false _*is given by the number of decoy positive PSMs in this search. This estimation method for two-pass searches is specified by the *two-pass estimation method*.

Second, in case in which the MSR step is applied, we first divide the set of spectra into two groups: *S*_1 _matched spectra in the first step and *S*_2 _remaining spectra. To estimate the false positives in the first pass, we use the single-pass estimation method with the spectra *S*_1 _instead of all the spectra. To estimate the false positives in the second pass, we use the two-pass estimation method with the spectra *S*_2 _instead of all the spectra. The final estimation of *N_false _*is given by summing up the two estimated numbers of the false positives.

The results of the four two-pass search methods are shown in Table 12.

###### Conclusion

For most cases, the two-pass searches produced significantly more PSMs than the single-pass search at the same factual FDR. The empirical FDR from traditional decoy database significantly underestimated the factual FDR, in particular when the MSR step was not used (shown in the search Y-10). On the other hand, the empirical FDR from the BK decoy database was close to the factual FDR, whether the MSR step was used or not (shown in the searches Y-12 and Y-13). The numbers of target PSMs in these searches were still larger than in the single-pass search. For example, MS-GFDB reported 3262 *- *2588 = 674 and 3103 *- *2588 = 515 additional PSMs in the searches Y-12 (without the MSR step) and Y-13 (with the MSR step), respectively, as compared to the search Y-1. The factual FDRs of these additional 674 and 515 PSMs were 1.8% and 1.4%, respectively. This indicates that the additional PSMs without the MSR step result in rather high FDR.

Based on the results, we recommend to use two-pass searches using the BK decoy database because it outputs more target PSMs than single-pass searches with reliable FDR estimation.

## Discussion

Reliable estimation of false discovery rates is a necessary precondition for the downstream utility of high throughput proteomics studies. Without accurate FDR estimates it is not possible to meaningfully compare results across different labs or search procedures and substantial amounts of time and resources may be wasted following 'surprising leads' later shown to be no more than just false positives. While the final decision of which FDR (e.g., 1% or 5%) is reasonable and appropriate for a particular experiment should ultimately rest with the researcher responsible for the analysis, it is important to be aware of the expected statistical consequences of the possible procedural choices to allow for both amelioration and critical evaluation of their effects in the resulting lists of identifications. Here we evaluated these possible effects using MS/MS data from samples where we were able to define a *factual *FDR estimator of 'true' FDR using strong indicators of false identifications that were not available to TDA or the database search engine.

While the particulars of specific experiments may warrant additional exploration, the results presented here indicate that the adoption of a simple set of guidelines could substantially improve the odds that TDA estimates of 'true' FDR will be within an acceptable interval around measured empirical FDRs. Conversely, we show that there are cases where PSM-level FDR is highly inappropriate since it results in a peptide-level FDR over 10*× *higher than the only reported FDR. In fact, we argue that peptide-level FDR should be the norm when reporting identification results and PSM-level FDR should be avoided whenever possible and require additional evidence from the authors showing that there are substantial reasons to avoid imposing peptide-level FDR. The main reason behind this strong assertion is that most MS based experiments are conducted with the purpose of identifying peptides and proteins for biological interpretation where one is not concerned about the identity of any particular spectrum but rather with the expected number of false positives in the list of identifications used for follow up analysis. Another reasonable way to control FDR is to impose protein-level FDR; however, these procedures usually faces difficulties of their own (e.g., how to handle peptides shared by multiple proteins) and should be addressed separately in a different study. Other aspects beyond the scope of this study that could also have a significant impact on the accuracy of TDA estimation of FDR are post-translational modifications, MS/MS acquisition modes (e.g., MS/MS + MS/MS/MS), local FDR (e.g., as used in PeptideProphet), spectral library searches [28], etc.

Out of the recommendations derived and supported by the results above, we observed that two-pass searches seem to be the most promising search strategy. Out of all tested strategies, two-pass searches came closest to identifying as many peptides as would be possible with perfect advance knowledge of the exact list of proteins in the sample of interest. Of course, it should be noted that such gains are likely to deteriorate for higher complexity samples where the second pass database is not substantially smaller than the initial database. Also we remark that the increased number of identified peptides does not necessarily mean the increased number of identified proteins in two-pass searches because the candidate proteins are fixed in the first pass of the searches.

## Competing interests

The authors declare that they have no competing interests.
